# MELD-score for risk stratification in cardiac surgery

**DOI:** 10.1007/s00380-023-02262-9

**Published:** 2023-04-01

**Authors:** Presheet Pathare, Mohamed Elbayomi, Michael Weyand, Colin Griesbach, Frank Harig

**Affiliations:** 1 Universitätsklinikum Erlangen, Herzchirurgische Klinik, Krankenhaus Straße, 12, 91054 Erlangen, Germany; 2 Chair of Spatial Data Science and Statistical Learning, Georg-August-Unversität Göttingen, Wilhelmsplatz, 1, 37073 Göttingen, Germany

**Keywords:** Preoperative, Cardiac surgery, Liver disease, MELD

## Abstract

The outcome of the patients undergoing cardiac surgery with cardiopulmonary bypass (CPB) is also influenced by the renal and hepatic organ functions. Risk stratification, using scores such as EURO Score II or STS Short-Term Risk Calculator for patients undergoing cardiac surgery with cardiopulmonary bypass, ignores the quantitative renal and hepatic function; therefore, MELD-Score was applied in these cases. We retrospectively examined patient data using the MELD score as a predictor of mortality. To perform a univariate analysis of the data, patients were classified into three groups based on the MELD Score: MELD < 10 (Group 1), MELD 10 to 19 (Group 2), and MELD ≥ 20 (Group 3). A total of 11,477 participants were included in the study, though several patients with either missing MELD scores or lack of creatinine, bilirubin, or INR levels were dropped from the original cohort. Eventually, 10,882 patients were included in the analysis. The primary outcome was defined as postoperative, in-hospital mortality. Secondary outcomes such as postoperative bleeding, including the requirement for repeat thoracotomy, postoperative neurological complications, and assessment of catecholamines on weaning from cardiopulmonary bypass/ requirement of mechanical circulatory support were examined. A higher MELD score was associated with increased postoperative mortality. Patients with MELD > 20 experienced a 31.2% postoperative mortality, compared to Group 1 (4.6%) and Group 2 (17.5%). The highest rates of postoperative bleeding (13.8%) and, repeat thoracotomy (13.2%) & postoperative pneumonia (17.4%) were seen in Group 3 (threefold increase when compared to Group 1, renal failure requiring dialysis (*N* = 235, 2.7% in Group 1 v/s *N* = 78, 22.9% in Group 3) or requiring high dose catecholamines post-operatively or mechanical circulatory support (IABP/ECLS). Incidentally, an increased MELD Score was not associated with a significant increase in the postoperative incidence of stroke/TIA or the presence of sternal wound infections/complications. A higher mortality was observed in patients with reduced liver and renal function, with a significant increase in patients with a MELD score > 20. As the current risk stratification scores do not consider this, we recommend applying the MELD score before considering patients for cardiac surgery.

## Introduction

The MELD Score was first described by Kamath, Malinchoc, Gordon et al. as a model to predict the outcomes of patients with portal hypertension undergoing Trans jugular intrahepatic portosystemic shunts (TIPS) [1], and was adopted by UNOS [2] to improve allocation times for patients waiting for a liver transplant [3]. Thus, the MELD Score established itself as a valid method to predict the survival of patients with end-stage liver disease [4]. The MELD Score has since also been used to assess the risk of patients with liver cirrhosis undergoing heart surgery. [5]. With the rising number of multimorbid patients undergoing cardiac surgery, preoperative risk stratification is becoming increasingly important.

Current risk stratification scores used for patients undergoing heart surgery include EUROSCORE II [6] and STS Short-Term Risk Calculator [7]. Although both scores are detailed and provide a reliable framework for risk assessment in patients undergoing heart surgery, their designs do not provide risk evaluation for patients with liver disease, depending on the severity of the disease. Liver disease has been shown to increase postoperative mortality after heart surgery by up to 22% [8] as an independent risk factor, and patients with cirrhosis have been shown to have higher rates of mortality [9].

Studies that evaluate MELD Score for risk stratification in patients undergoing cardiac surgery focus on outcomes in patients with cirrhosis. However, patients with liver disease, which has not progressed to its end stage, also have increased mortality and need further assessment. Due to the various synthetic functions of the liver, which are critical to the postoperative period, postoperative hepatic dysfunction results in increased postoperative morbidity and mortality. [10].

In the present study, the authors have reviewed retrospectively the MELD Score of patients undergoing heart surgery from 2000 to 2019 and the mortality in these patients after all-type cardiothoracic operations with cardiopulmonary bypass. The aim of the study was to develop a risk model to predict mortality in surgical patients with respect to their MELD Score.

## Methods

**Fig. 1:**
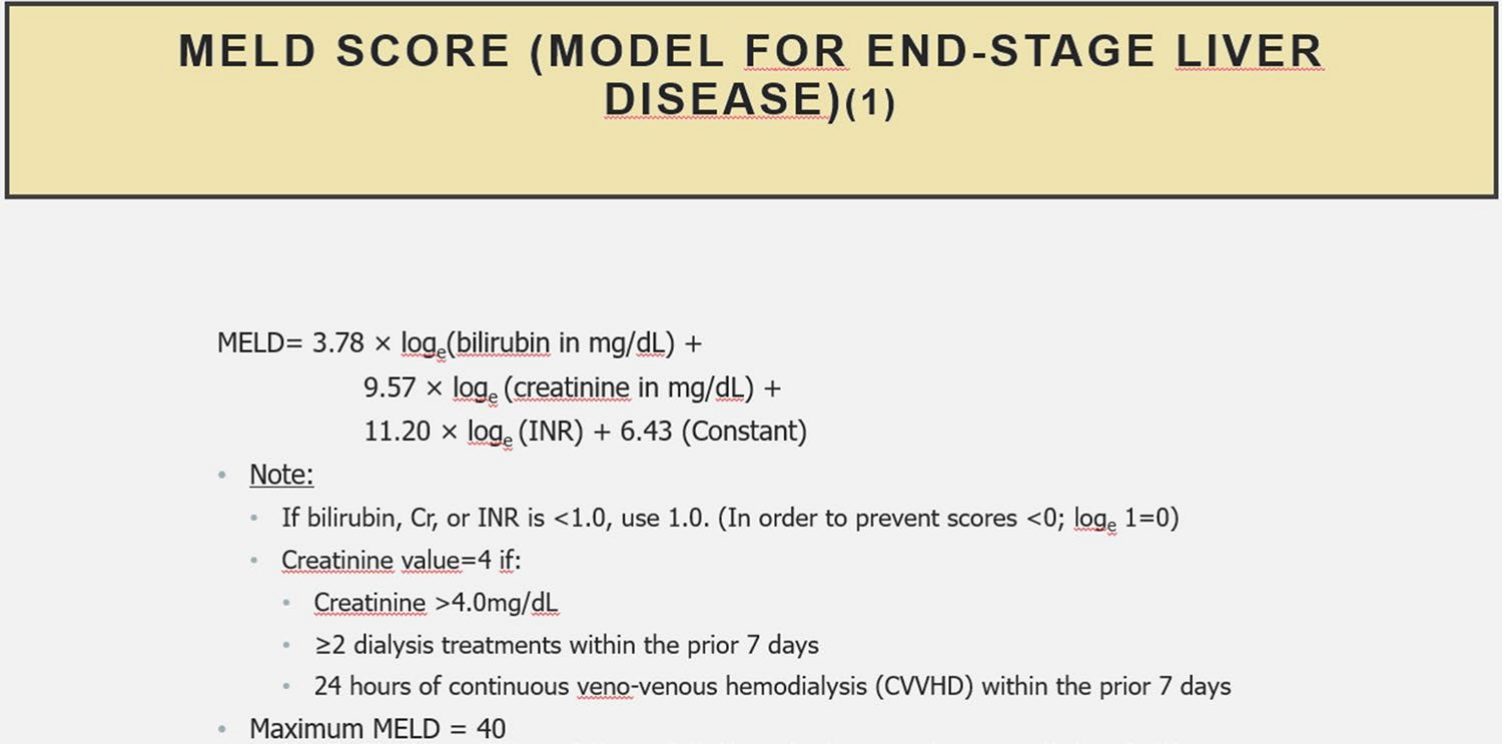
Formula for the calculation of MELD Score

From 1 January 2000 through 2019, 11,434 adult patients with all-type heart disease underwent cardiac surgery with median sternotomy using the heart–lung-machine at the authors’ institution, a quaternary, acute care university hospital in northern Bavaria. Inclusion criteria for patients included the following: cardiac surgery with median sternotomy and use of cardiopulmonary bypass. The MELD Score of the patients was calculated using the table and formula shown in Fig. 1. Patients who underwent urgent or emergent procedures or a redo procedure were also included. Patients whose bilirubin, creatinine, or INR could not be determined pre-operatively were excluded from the study.

This retrospective study complied with the Helsinki Declaration (2000), and approval to perform this analysis was given by the local Ethics Committee (EC, No.22–261-Br), based on the model of the study. Therefore, the informed consent of the patients was waived by the ethics committee.

### Definitions

The MELD score was calculated using the formula published by Kamath et al. [1]. To perform a univariate analysis of the data, patients were classified into three groups based on the MELD Score: MELD < 10 (Group1), MELD 10 to 19 (Group 2), and MELD ≥ 20 (Group 3).

### Statistical analysis

Categorical data are shown as counts (percentages) and continuous variables as mean SD or median with interquartile ranges in brackets. The χ2 test was used for categorical variables, and the independent t-test or Anova test for continuous variables.

Generalized linear mixed models of relevant variables were used to calculate the correlation between MELD and surgical outcomes and postoperative variables. Using linear logarithmic models, the projected EURO SCORE for the desired outcome was employed first, followed by MELD with the primary outcome as postoperative all-cause mortality and various secondary outcomes. The change in area under the curve (AUC) for the nested models, the variable effect size (Odds ratio), and other metrics were used to evaluate the extra variables' usefulness.

Further analysis was conducted for patients with a MELD Score of 20 or more to assess the preoperative risk factors contributing to all-cause mortality, and results were expressed as adjusted odds ratios, with CI between 2.5 and 97.5%.

A total of 11,477 participants were included in the study, though several patients with either missing MELD scores or lack of creatinine, bilirubin, or INR levels were dropped from the original cohort. Eventually, 10,882 patients were included in the analysis.

The primary outcome was defined as postoperative, in-hospital mortality. Secondary outcomes such as postoperative bleeding, including requirement for repeat thoracotomy, postoperative neurological complications, and assessment of catecholamines on weaning from cardio-pulmonary bypass/requirement of mechanical circulatory support were examined as secondary outcomes.

#### Risk adjustment, MELD, EURO score

To assess how the MELD Score can additionally improve the risk assessment of the EURO Score, a logistic regression analysis was performed.

First, for each outcome variable of interest, logistic regression with respect to EURO SCORE alone was performed. This was used to derive the Base AUC value, i.e., the prognostic quality of the EURO Score for a particular outcome variable alone (e.g., the ability of the EURO Score to predict postoperative mortality). Then, for each outcome, another logistic regression model was estimated, which additionally included MELD as an independent predictor. This comparison is shown as the improved AUC values.

Using both values, an Odds ratio with 95% CI was derived to show the comparison of MELD to the EURO Score.

#### Statistical analysis of preoperative factors influencing morbidity in Group 3

In an effort to further improve the preoperative risk assessment, further analysis of patients with a MELD Score > 20 was conducted, to examine if certain conditions were risk factors for postoperative mortality. Odds ratios for the individual variables as preoperative risk factors were calculated with the primary outcome of postoperative mortality in patients with MELD > 20 relative to patients with lesser MELD Scores. In these patients, risk factors such as preexisting liver diseases, presence of endocarditis, patients presenting with myocardial infarction and patients with chronic obstructive lung disease have a moderate or severe effect on the outcome with increased Odds ratios.

Furthermore, our analysis found that the preoperative presence of dialysis, stroke, diabetes, or hyperlipidemia did not contribute significantly to mortality in patients with an increased MELD Score.

## Results

### Patients’ initial characteristics and operational variables

The majority of the included patients (*N* = 8840, 81.2%) had a low MELD score of less than 10, whereas 15.64% had a moderately high MELD of between 10 and 19, and 3.12% had a significantly increased MELD of greater than 20. Distributions of comorbidities and demographics are shown in Table 1.

**Table 1:** Demographic data of all patients analyzed, divided into groups based on their MELD Scores

MELD Score	< 10 (*N* = 8840)	10–19 (*N* = 1702)	> = 20 (*N* = 340)	*P* value
Preoperative risk factors				
Age mean(SD) (years)	67.049 (10.751)	68.449 (11.375)	61.347 (11.771)	< 0.001
Sex	2742 (31.0%)	363 (21.4%)	69 (20.3%)	< 0.001
Height mean(Sd) (cm)	169.808 (8.903)	171.629 (8.780)	173.453 (9.094)	< 0.001
Weight mean(SD) (kg)	81.272 (15.235)	82.939 (16.238)	83.424 (17.280)	< 0.001
COPD	805 (9.1%)	193 (11.3%)	41 (12.1%)	0.004
Apoplex	552 (6.2%)	153 (9.0%)	36 (10.6%)	< 0.001
Diabetes	2662 (30.3%)	632 (37.5%)	126 (37.5%)	< 0.001
Dialysis	3227 (36.5%)	581 (34.1%)	179 (52.6%)	< 0.001
Hyperlipidemia	6731 (76.7%)	1181 (70.5%)	187 (56.8%)	< 0.001
Endocarditis	195 (2.2%)	90 (5.3%)	43 (12.6%)	< 0.001
Cardiogenic shock	159 (1.8%)	160 (9.4%)	62 (18.2%)	< 0.001
Peripheral arterial disease	913 (10.3%)	216 (12.7%)	42 (12.4%)	0.010
Myocardial infarction	2539 (28.7%)	557 (32.7%)	109 (32.1%)	0.002
Preoperative reanimation	137 (1.6%)	73 (4.3%)	32 (9.4%)	< 0.001
Euroscore 2 mean(SD)	6.011 (3.171)	8.509 (3.714)	9.768 (3.725)	< 0.001

An increased prevalence of concomitant conditions, such as diabetes (*N* = 126, 37.5%), chronic obstructive lung disease (*N* = 41, 12.1%), and the presence of endocarditis (*N* = 43, 12.6%)) was observed in patients in Group 3. There was also a high number of patients in Group 3 who required preoperative dialysis (*N* = 179, 52.6%).

The operative data of the patients included in the analysis are shown in Table 2.

**Table 2:** Operative characteristics of all patients analyzed, sorted by the respective MELD Scores

MELD Score	< 10 (*N* = 8840)	10–19 (*N* = 1702)	> = 20 (*N* = 340)	*P* value
Operation				
CABG	6645 (75.7%)	983 (60.6%)	134 (45.1%)	< 0.001
LVAD	38 (0.4%)	87 (5.4%)	62 (20.9%)	< 0.001
Value	1608 (18.3%)	439 (27.0%)	85 (28.6%)	< 0.001
Cardiac tumor	79 (0.9%)	16 (1.0%)	1 (0.3%)	< 0.001
Aortic surgery	347 (4.0%)	87 (5.4%)	13 (4.4%)	< 0.001
Congenital Anomaly in Adults	58 (0.7%)	8 (0.5%)	2 (0.7%)	< 0.001
Urgency				
Elective	5187 (58.7%)	751 (44.1%)	97 (28.5%)	< 0.001
Urgent	3074 (34.8%)	657 (38.6%)	145 (42.6%)	< 0.001
Emergency	579 (6.5%)	294 (17.3%)	98 (28.8%)	< 0.001
Cross clamp time (in min) Mean(SD)	49.262 (26.147)	52.078 (36.172)	48.868 (46.763)	< 0.001

Average EURO Scores in each group were as follows: Group 1: 6.011 (SD-3.171), Group 2: 8.509 (SD-3.714), and Group 3 9.768 (SD-3.725). As such, an increase in MELD Score was seen to be associated with an increased EURO Score (*p* < 0.001).

The MELD Score is shown to be more sensitive when compared to the EURO Score in predicting certain postoperative outcomes (e.g., postoperative mortality; AOR of 1.09 per MELD score point; need for dialysis; AOR, 1.1 per MELD score point, pneumonia, etc. *p* < 0.0001). Both scores, however, have equal predictive capabilities with respect to postoperative stroke/TIA, sternal wound complications, and the need for IABP after cross-clamping (AOR 1–1.02 per MELD score point) (Table 3).

**Table 3:** Comparison of MELD Score and EURO Score II with respect to postoperative outcomes.

Categories	AOR	95% CI	*p* Value	Base AUC	AUC
In-Hospital mortality	1.09	1.07–1.1	0	0.785	0.811
Occurance of postoperative complication	1.06	1.05–1.07	0	0.682	0.693
Postoperative bleeding	1.05	1.03–1.06	0	0.659	0.674
Rethoracotomy	1.04	1.03–1.05	0	0.664	0.676
Pneumonia	1.04	1.03–1.05	0	0.666	0.684
Sternal wound complications	1.01	0.99–1.04	0.22	0.528	0.531
postoperative apoplex	1.01	0.97–1.06	0.578	0.65	0.652
TIA	1	0.93–1.07	0.976	0.572	0.57
Intermittant hemofiltration	1.1	1.09–1.12	0	0.753	0.806
Permanent dialysis	1.16	1.15–1.18	0	0.739	0.856
Postoperative ECMO	1.06	1.03–1.09	0	0.798	0.808
Exitus intabula	1.08	1.03–1.12	0	0.901	0.925
Postoperative IABP	1.02	1.01–1.03	0.002	0.68	0.685
Postoperative high dose catecholamines	1.06	1.03–1.09 0	0	0.798	0.808

*AOR* adjusted odd’s ratio, *AUC* area under the curve

A proportional rise in primary and secondary outcomes was seen in Groups 2&3 as compared to patients from Group 1 with a low MELD Score.

**Fig. 2:**
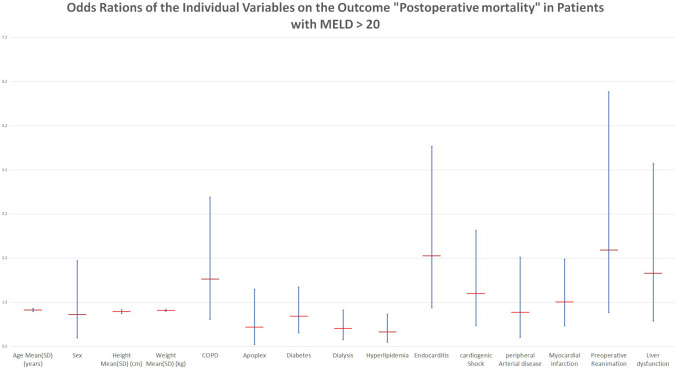
Odds Ratios of the Individual Variables on the Outcome “Postoperative mortality” in Patients with MELD > 20. Preoperative risk factors with their CI are plotted on a logarithmic scale

Patients with MELD > 20 experienced a 31.2% postoperative mortality, compared to Group 1 (4.6%) and Group 2 (17.5%) (Table 4). The highest rates of postoperative bleeding (13.8%) and, repeat thoracotomy (13.2%) & postoperative pneumonia (17.4%) were seen in Group 3 (threefold increase when compared to Group 1, renal failure requiring dialysis (*N* = 235, 2.7% in Group 1 v/s *N* = 78, 22.9% in Group 3) or requiring high dose catecholamines post-operatively or mechanical circulatory support (IABP/ECLS). These results are summarized in Fig. 2 & Table 5.

**Table 4:** Occurrence of postoperative complications according to MELD Score

MELD score	< 10 (*N* = 8840)	10–19 (*N* = 1702)	> = 20 (*N* = 340)	*P*-value
Outcomes				
In-Hospital mortality	406 (4.6%)	298 (17.5%)	106 (31.2%)	< 0.001
Postoperative hospital stay Mean(SD) (days)	13.688 (10.377)	19.056 (19.476)	22.065 (22.697)	< 0.001
Postoperative complications	6277 (71.0%)	1465 (86.2%)	310 (91.2%)	< 0.001
Bleeding	351 (4.2%)	128 (7.8%)	45 (13.8%)	< 0.001
Rethoracotomy	436 (4.9%)	179 (10.5%)	45 (13.2%)	< 0.001
Pneumonia	557 (6.3%)	234 (13.7%)	59 (17.4%)	< 0.001
Sternal wound complications	218 (2.5%)	66 (3.9%)	7 (2.1%)	0.003
postoperative apoplex	39 (0.4%)	13 (0.8%)	3 (0.9%)	0.139
TIA	20 (0.2%)	7 (0.4%)	1 (0.3%)	0.382
Intermittant hemofiltration	235 (2.7%)	206 (12.1%)	78 (22.9%)	< 0.001
Permanent dialysis	195 (2.2%)	262 (15.4%)	140 (41.2%)	< 0.001
Postoperative ECMO	37 (0.4%)	23 (1.4%)	11 (3.2%)	< 0.001
Postoperative IABP	598 (6.8%)	226 (13.3%)	42 (12.4%)	< 0.001
Postoperative high dose catecholamines	37 (0.4%)	23 (1.4%)	11 (3.2%)	< 0.001
Exitus intabula	8 (0.1%)	15 (0.9%)	6 (1.8%)	< 0.001

**Table 5:** Risk factors and their influence on mortality for patients with MELD > 20

Categories	Odd’s Ratio	2.5% CI of OR	97.5% CI of OR	*p*.Werte
Age mean(SD) (years)	1.03	1.00	1.06	0.0238
Sex	0.93	0.40	2.14	0.8737
Height mean(SD) (cm)	0.99	0.95	1.03	0.5743
Weight mean(SD) (kg)	1.02	1.00	1.04	0.0235
COPD	1.73	0.82	3.58	0.1433
Apoplex	0.64	0.25	1.49	0.3184
Diabetes	0.89	0.51	1.54	0.6831
Dialysis	1	0.36	1.02	0.0613
Hyperlipidemia	0.5	0.30	0.93	0.0265
Endocarditis	2.26	1.08	4.73	0.0304
cardiogenic shock	1.40	0.68	2.83	0.3555
peripheral arterial disease	0.98	0.41	2.22	0.9661
Myocardial infarction	1.21	0.67	2.18	0.5308
Preoperative Reanimation	2.39	0.97	5.97	0.0584
Liver dysfunction	1.86	0.78	4.34	0.1531

Incidentally, an increased MELD Score was not associated with a significant increase in the postoperative incidence of stroke/TIA or the presence of sternal wound infections/complications.

## Discussion

Calculating the MELD score can have a profound impact as a tool for determining the postoperative outcome. Higher MELD scores are associated with greater comorbidities which influence the postoperative outcome.

When stratifying patients, especially those with existing liver disease or the subjectively reduced general condition of patients, current risk stratification strategies with scores such as STS Short-Term Risk Calculator or EURO Score are severely constrained. The MELD score, in contrast, employs three straightforward measures to characterize the level of liver disease, directly measuring it with INR and bilirubin as well as any related renal impairment.

As observed above, an increased MELD Score has a significant impact on postoperative bleeding and other complications. Patients with a MELD of more than 20 are particularly affected. These results should be viewed critically, as a higher percentage of patients in this group underwent emergency surgery when compared to the other groups (Table 2). Factors responsible for an increased MELD Score (renal insufficiency, liver failure, and its various causes, etc.) are also responsible for cardiovascular disease and the emergencies arising therefrom. However, acute cardiovascular emergencies could lead to an acute increase in MELD Scores preoperatively. Further study of long-term pre- and postoperative data may help to differentiate this.

Due to the myriad of functions and biosynthetic capabilities of the liver, unlike other organ systems (dialysis, ECLS), there lacks, to date, a clinically applicable organ replacement therapy in cases of terminal liver disease. Whether the development of such liver support systems could be translated into a survival benefit in the future is still the presence of liver disease has been proven to be an independent risk factor for the postoperative outcome [5]. On further analysis of patients with preexisting liver cirrhosis, it is apparent that the MELD score is a more valid method to assess risk [8].

Though the MELD score can be used as a standalone tool to determine post-operative outcomes, it can be used to enhance existing risk stratification strategies. Through the above analysis, it has been shown that MELD continues to be a substantial independent predictor of operational mortality even after risk adjustment, which enhances the effectiveness of the EURO Score. Although it can also predict significant morbidity and associated problems, its predictive power is not applicable to all avenues of postoperative complications.

Cardiopulmonary bypass duration has been shown to be an independent predictor of morbidity and mortality after cardiac surgery [12]. As end-organ dysfunction, which plays an important role in postoperative outcomes, is adversely affected by cardiopulmonary bypass and cross-clamp times, scores such as MELD can help determine which patients are particularly predisposed to adverse operative outcomes.

Uncertain, given the scarcity of available results of RCTs. [11].

The complexity of liver function also justifies the observations obtained. Hemostasis and the production of various proteins, including antibodies, are important functions of the liver. Using the MELD Score as an indicator of liver function, we can see that the increased MELD Score shows an increased incidence of postoperative pneumonia & bleeding complications. Since the MELD Score also accounts for renal function, it follows that it directly correlates to the incidence of postoperative hemofiltration and the requirement for dialysis. Results that do not show a significant difference between the groups are less intuitive to discern. Wound healing complications and incidence of cerebrovascular events are multifactorial. STS and EURO Scores take into account risk factors that may influence the incidence of these complications (presence of COPD, diabetes on insulin, peripheral/central arterial disease, etc.). The MELD Score does not provide any benefit when trying to assess the incidence of these particular complications; it does, however, show improved prediction capability when compared to EURO Score when assessing other complications, as shown in Table 3 (AUC and Base AUC).

Liver dysfunction, when present preoperatively, is an important risk factor for postoperative mortality. In patients with a MELD Score of > 20, with an OR of 1.86, the presence of liver dysfunction is a reliable, independent indicator of postoperative mortality. A substantial subset of patients presented with and required therapy for cardiogenic shock (mech. circulatory support, LVAD, etc.) (Table 2). Due to the pathophysiology of these diseases, they may cause liver congestion, and a reduction in its function, and therefore, these patients have higher MELD Scores (MELD > 20) on presentation.

Certain patients may present with diseases and symptoms that mask those of liver insufficiency. Furthermore, disruption of liver and renal function may arise from certain cardiac pathologies (e.g., right heart failure, terminal heart failure requiring LVAD, etc.). Evaluation of these patients is performed using more appropriate scores (INTERMACS, Seattle Heart Failure model, etc.). It is worthwhile to compare these models in a prospective study setting and supplement our understanding of liver function using translational research.

The current benchmark for liver transplantation, MELD-Na, may be calculated by assessing preoperative sodium levels. The easy integration of sodium levels and INR along with bilirubin in the ongoing automatic computation of the overall score would not necessitate any retroactive adjustments because the new EURO score can be scaled to meet MELD.

The use of scores for risk assessment in preoperative patients gives a reliable statistical base for the prediction of mortality. However, caution should be exercised in the interpretation of such results in specific clinical scenarios, as the circumstances of the individual patient are not always considered and could require the precise judgment of the clinician.

## Limitations

Due to the retrospective, single-center nature of the study, it carries all the limitations that are associated with such models, including the inability to prove causation. During the time under consideration, there may have been changes that were not accounted for, e.g., changes in laboratory methods for measurement of bilirubin, serum creatinine, changes in operative standards, changes in prostheses, etc.

Given that both MELD and the EURO score utilized for risk adjustment incorporate creatinine, there is some overlap in the evaluation that likely understates the influence of MELD in the factors that rely on this value.

Operative mortality is not only dependent on the preoperative state of the patient, but also on the post-operative complications. In a few cases, there may have been unforeseen circumstances that were the cause of the mortality but were not associated with preoperative diagnoses.

Changes were made to the EUROSCORE and STS Short-Term Risk Calculator Models during the period under study.

## Conclusion

MELD Score can accurately predict the outcome particularly in high-risk patients undergoing heart surgery with cardiopulmonary bypass, especially considering that current risk stratification models used in cardiac surgery do not take into account liver function.
